# Association Between Sleep Position, Obesity, and Obstructive Sleep Apnea Severity

**DOI:** 10.3390/jpm14111087

**Published:** 2024-11-01

**Authors:** Mia Strohm, Amro Daboul, Anne Obst, Antoine Weihs, Chia-Jung Busch, Thomas Bremert, Jochen Fanghänel, Tatyana Ivanovska, Ingo Fietze, Thomas Penzel, Ralf Ewert, Markus Krüger

**Affiliations:** 1 Department of Prosthodontics, Gerodontolgy and Biomaterials, University Medicine Greifswald, 17489 Greifswald, Germany; daboula@uni-greifswald.de (A.D.); markuskr@uni-greifswald.de (M.K.); 2 Department for Internal Medicine B, University Medicine Greifswald, 17489 Greifswald, Germany; anne.obst@uni-greifswald.de (A.O.); ralf.ewert@med.uni-greifswald.de (R.E.); 3 German Center for Neurodegenerative Diseases (DZNE), 17489 Greifswald, Germany; antoine.weihs@med.uni-greifswald.de; 4 Department of Psychiatry and Psychotherapy, University Medicine Greifswald, 17489 Greifswald, Germany; 5 Department of Otorhinolaryngology, University Medicine Greifswald, 17489 Greifswald, Germany; chia-jung.busch@med.uni-greifswald.de (C.-J.B.); thomas.bremert@med.uni-greifswald.de (T.B.); 6 Department of Orthodontics, Dental School, University of Regensburg, 93053 Regensburg, Germany; jochen.fanghaenel@klinik.uni-regensburg.de; 7 Ostbayerische Technische Hochschule Amberg-Weiden, Fakultät für Elektrotechnik, Medien und Informatik, 92224 Amberg, Germany; t.ivanovska@oth-aw.de; 8 Sleep Center, University Hospital Charité Berlin, 10117 Berlin, Germany; ingo.fietze@charite.de (I.F.); thomas.penzel@charite.de (T.P.)

**Keywords:** obstructive sleep apnea, OSA, sleep position, BMI, Apnea-Hypopnea Index, AHI, sleep disordered breathing, population based

## Abstract

Background: This study examines the relationship between obstructive sleep apnea severity, sleep position, and body weight, particularly focusing on the negative impact of sleeping in a supine position combined with being overweight in a population-based sample. Methods: The Apnea-Hypopnea Index (AHI) was utilized as a marker of OSA severity and sleep position from a standardized overnight polysomnography. Participants were categorized by body mass index (BMI) (kg/m^2^) into normal weight/underweight (<25) and overweight (≥25). Results and Conclusions: The results indicated a higher mean Apnea-Hypopnea Index for those sleeping in the supine position compared to other positions, with overweight individuals experiencing a proportionally greater impact from sleep position than their normal-weight counterparts.

## 1. Introduction

Obstructive sleep apnea (OSA) is one of the most common sleep disorders, with an estimated prevalence from 9 to 38% in the worldwide adult population [1,2]. OSA is characterized by recurrent events of partial or complete collapse of the upper airways, causing frequent episodes of apnea and hypopnea. Its symptoms include sleep fragmentation, excessive daytime sleepiness, and depression, which can lead to a significant decline in quality of life [3,4,5] and predispose an individual to a number of chronic cardiovascular and metabolic disorders [6,7]. The severity of OSA is commonly assessed using the Apnea-Hypopnea Index (AHI), which measures the frequency of apneas and hypopneas per hour of sleep [8]. One of the most frequently encountered risk factors that notably impacts the severity of OSA is a high body mass index (BMI). Also particularly affected are middle-aged or older men, postmenopausal women, people with specific abnormalities of the bony and soft tissue structure of the head and neck, smokers, and children with large tonsils or adenoids, as well as adults and children with Down syndrome [9,10,11].

While the exact mechanisms of the disorder remain largely unclear, it is suggested that the pathophysiology is multifactorial [12,13]. This might involve abnormalities in oropharyngeal anatomy, such as variations in the upper-airway diameters between patients with and without OSA. Patients with OSA tend to have smaller upper-airway diameters compared to those without patients with OSA. Additionally, the retrograde position of the lower jaw relative to the upper jaw contributes to a reduced size of the upper airways, leading to increased collapsibility [13,14,15]. In individuals with a high BMI, the reduced lung volume and the enlarged parapharyngeal fat pads decrease the traction on the lower trachea, potentially promoting a collapse in the pharyngeal area [16,17].

Sleep position has a significant impact on the severity, frequency, and duration of apneas, with most patients having a lower AHI in a non-supine position (i.e., sleeping on their left or right side) than in the supine position [14,18,19,20]. Positional dependence can be determined by questionnaires [19] or positional sensors during a polysomnography (PSG) examination [21,22].

A possible explanation for the positional dependency is the anatomical structures that change in the supine position, even though the exact mechanism is largely unclear. It has been shown using the acoustic reflection technique that the pharyngeal airway becomes smaller in the supine position [21,22,23]. Additionally, the tongue base, epiglottis, and soft tissue show obstruction in the supine position, which improves when the sleep position changes from supine to lateral, as shown by drug-induced sleep endoscopy, although the exact mechanism is not fully clear [24].

In some studies involving patients with OSA, an interconnection between the impact of BMI and sleeping position on the AHI has been described [25,26,27]. Itasaka et al. (2000) [25] found that normal-weight patients benefited more from a non-supine sleeping position regarding the parameters AHI, intraesophageal pressure, and the lowest oxygen saturation than with overweight and obesity. Joosten et al. (2017) [26] studied the consequence of weight loss due to dieting or surgical intervention on the AHI. They found that more patients benefited from a non-supine sleeping position after weight loss than before. Furthermore, changes in AHI were more closely related to changes in body weight when measured in a non-supine sleeping position. This effect was even more pronounced in the patients that received the surgical intervention, which led to an overall higher weight loss compared to the patients who were dieting. Therefore, in our current study, we examine the connection between the severity of OSA and the sleeping position in a population-based sample. Alongside the well-established better outcomes in non-supine sleeping positions, we expect a significant interaction between sleep position and overweight in influencing the severity of OSA as measured by the AHI: the impact of sleeping in a supine position on the AHI should be more pronounced among individuals with overweight compared to those with normal or lower weight.

## 2. Materials and Methods

### 2.1. Sample

A subsample of the Study of Health in Pomerania (SHIP) was used for our study. SHIP is a randomly sampled population-based cohort study designed to investigate various health variables among the residents of Pomerania and monitor their development over time [28]. We exclusively utilized comprehensive data sets, comprising 1209 individuals with complete AHI data of SHIP-Trend-0. Out of these, position data was accessible for 1097 people. Participants with implausible position data (i.e., sleeping more than 15 min in an upright position was considered as an ongoing equipment malfunction) were excluded, leaving 1045 complete data sets.

The controlled and standardized polysomnography (PSG) adhered to the guidelines set by the American Academy of Sleep Medicine (AASM) [29]. Various sensors were strategically placed on the participants’ bodies to ensure a comprehensive sleep analysis. The sensors monitored body movements, respiratory airflow, arterial blood oxygen saturation, and snoring patterns. As confounders, we used age (in years), BMI (kg/m^2^), and sex (male/female). For the analysis of an interaction effect, participants with a BMI under 25 were considered normal weight or underweight and a BMI of 25 or above as overweight. See Table 1 for an overview.

### 2.2. Sleep Data

#### 2.2.1. Overnight PSG

We conducted an overnight polysomnography in a laboratory setting, following the guidelines of the American Academy of Sleep Medicine (AASM). ALICE 5 devices from Philips Respironics (Philips Respironics, Eindhoven, The Netherlands), were used for this study. The recording setup included six electroencephalogram (EEG) channels, two electrooculogram (EOG) channels, two electromyogram (EMG) channels positioned at the chin and tibialis muscles, one electrocardiogram (ECG) channel, respiratory inductive plethysmography, a nasal pressure sensor, pulse oximetry, a microphone for snoring detection, and a body position sensor.

#### 2.2.2. Sleep Position

Sensors (body position sensor for ALICE 5 by Philips Respironics) were attached to the patients during the overnight polysomnography (PSG) to determine their body position. In this context, the supine position, lateral position, prone position, and upright position could be measured. This resulted in the categorization as supine, prone, left, and right sleeping position. Sleeping in an upright position was discarded as a measurement error. To reduce the complexity of our final analyses, the determination of sleep position was narrowed down to percent of sleep spent in the supine position.

### 2.3. Outcome AHI

We used the AHI as our outcome, which is calculated by dividing the total number of apneas and hypopneas by the total sleep study duration. With the help of the AHI, we can estimate the severity of OSA. An AHI under 5 is normal, between 5 and 15 is mild, between 15 and 30 is moderate, and over 30 is severe, as can be seen in Table 1.

### 2.4. Data Analyses

Data analysis and visualization were conducted using R Statistics (2023.09.0 Build 463) and splines [30] and the packages haven [31], dplyr [32], tidyr [33], MASS [34], ggplot2 [35], olsrr [36], and quantreg [37]. Ordinal logistic regression was conducted to test the general impact of sleeping in a supine position and multiple linear regression to highlight an interaction with body weight. Models are specified in detail in Section 3.

## 3. Results

### 3.1. Descriptive Analysis

**Figure 1:**
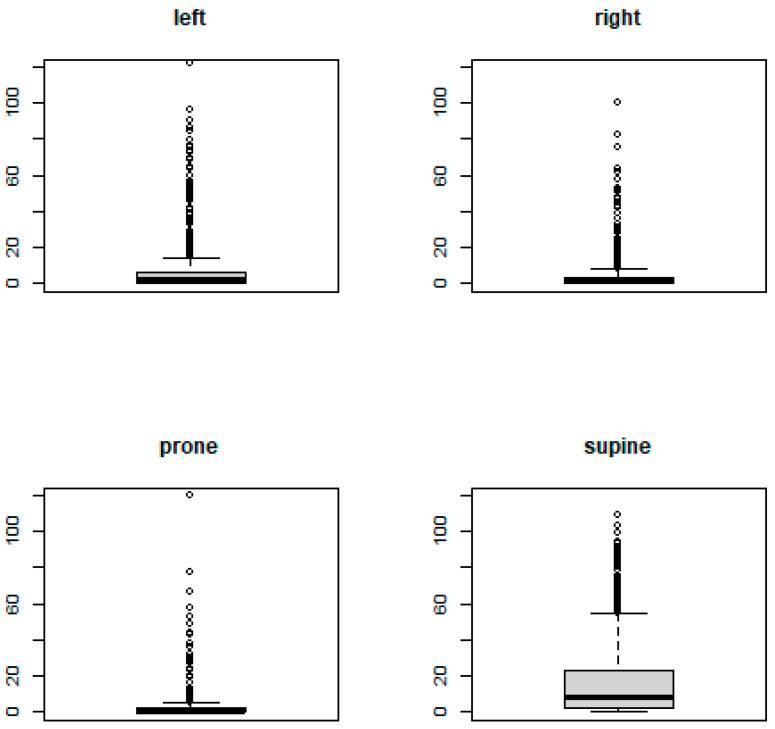
Boxplots for the AHI measured in the different sleeping positions.

The mean AHI was measured for participants sleeping in a supine position, left or right side, or in a prone position. Descriptively, the mean AHI in the supine position (*m* = 16.7, SD = 21.1) deviated from the other positions (left side: *m* = 6.7, SD = 14.6, right side: *m* = 4.1, SD = 10.3, prone: *m* = 4.8, SD = 13.8). As can be seen in Figure 1, boxplots show a large number of data points beyond the upper whiskers (1.5 times the interquartile range from the 75th percentile). These apparent outliers and the high standard deviations were to be expected, as roughly half of the participants had a healthy AHI of below 5 while there were relatively few participants with a severely high BMI above 29 (see Table 1). The boxplot for the supine position indicates a greater number of cases with mild and moderately high AHI than the boxplots for all other positions.

**Figure 2:**
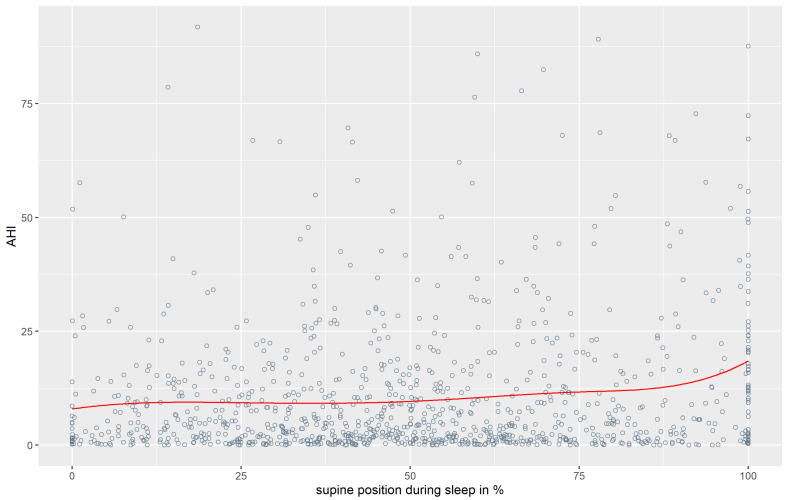
Data points and splines (knots at 25, 50, 75) for Sleep Position and AHI.

For further analysis, the complexity of the data was reduced. For the variable sleep position, we used the percent of sleeping time participants spent in the supine position (see Section 2). A visual inspection of the splines, which can be seen in Figure 2, indicates a close to linear relationship between Sleep Position and BMI. The longer participants spent in the supine position, the higher their AHI.

**Figure 3:**
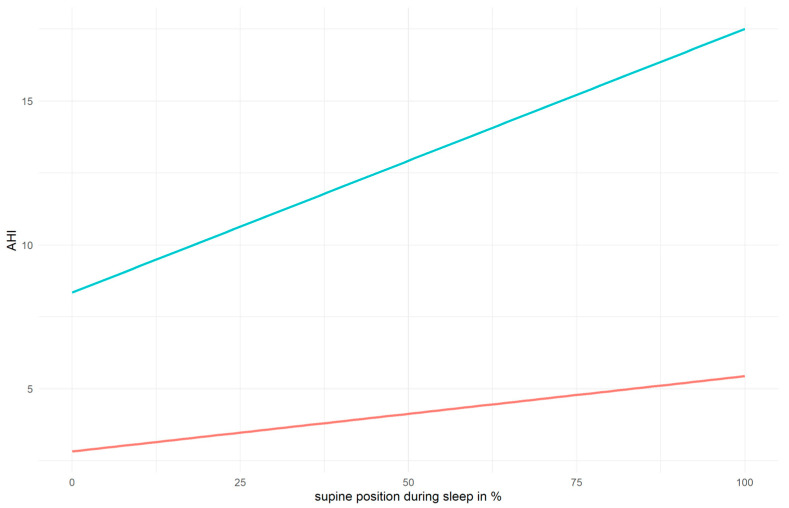
Linear functions of sleep position on the AHI for the different weight groups (turquoise: overweight, red: normal weight or underweight).

To visualize and subsequently test the assumed interaction between sleep position and BMI, participants were categorized as normal weight or underweight (BMI below 25) on the one hand and as overweight (BMI 25 or above) on the other (Figure 3). The linear trends between the sleep position and AHI indicated a steeper slope for the overweight (*a* = 0.092) group compared to the normal-weight and underweight group (*a* = 0.026).

### 3.2. Ordinal Logistic Regression Analysis

Initially, we assessed the potential impact of the sleeping position on the AHI. To achieve this, an ordinal logistic regression model with the continuous risk indicator sleep position (percent of time participants spent sleeping in a supine position) and the confounders age (in years), BMI (kg/m^2^), and sex (male/female) on AHI severity (AHI < 5: normal, 5 ≤ AHI < 15: mild, 15 ≤ AHI < 30: moderate, AHI ≥ 30: severe) was computed. The ordinal logistic regression was chosen, as we aimed to assess the relationship between the severity of sleep apnea, calculate odd ratios (ORs), and account for the non-normal distribution of the AHI.

For the risk indicator sleep position a, significant effect, *p* < 0.001, OR 1.02 (CI 1.01; 1.02), was found, indicating 2% higher chance of being in the next higher severity grade per every percent point spent extra sleeping in a supine position.

### 3.3. Multiple Linear Regression Analysis

Subsequently, we tested the proposed interaction between the participants’ sleeping position and their weight. Therefore, a multiple linear regression with the predictors sleep position (percent of time participants spent sleeping in a supine position), Overweight (yes/no), and the confounders age (in years) and sex (male/female) on the continuous outcome AHI was computed. This yielded a significant effect for the interaction between sleep position and overweight (*p* < 0.01), being in line with the hypothesis that overweight participants are more impacted by sleeping in a supine position than normal-weight and underweight ones (see Figure 3). (This interaction calls into question the interpretability of the main effect observed in the ordinal logistic regression analysis above. Consequently, the analysis was repeated without the confounding variable BMI, separately for each weight group. This yielded significant effects for both overweight participants (*p* < 0.001, OR 1.02 (CI 1.01; 1.02)) and the normal-weight and underweight ones (*p* = 0.04, OR 1.01 (CI > 1; 1.03)), substantiating that on a group level, AHI was lower when participants were sleeping on their side.)

A non-normal distribution of the outcome, and, more importantly, a non-normal distribution of the residuals from the analysis, can potentially violate the assumptions in linear regression. However, our relatively large sample might have mitigated such violation [38]. Nevertheless, additionally, a quantile regression analysis with the same variables for τ = 0.25 was computed, yielding the same results concerning the interaction.

## 4. Strength and Limitations

Our study’s primary advantage lies in its design, which is based on the general population with the utilization of the SHIP-Trend cohort, including 1045 participants, as opposed to smaller studies with a limited number of patients. This large-scale approach positions our findings as particularly robust and well suited for drawing comprehensive conclusions in the realm of sleep research.

Also contributing to this study’s validity were the quality and standardization of the examinations in SHIP. To ensure an objective measurement of sleep, we utilized PSG, acknowledged as the gold standard in sleep monitoring, as in other studies [22,39]. This approach allowed us to minimize reliance on subjective questionnaires. There were differences in how sleep data were gathered across the studies. In contrast to other studies, such as the one conducted by Cerritelli et al. (2022) [19], which determined sleep position through questionnaires and partner interviews, our method is considered more reliable.

On the other hand, the limitations come from the simplified the model by (a) distinguishing between the degree of time in supine and non-supine position only and (b) categorizing participants into overweight and non-overweight groups to present our results clearly. Furthermore, our study cannot provide information on how the AHI would change if the supine position was avoided because sleep position was not randomized but chosen freely by the participants themselves. The retrospective nature of our study and only one measuring point limit the interpretation of causality. While no indication for a relevant bias was found in previous studies concerning OSA and our data set, participants with sleeping disorders or sleeping trouble might be over-represented in our sample [10,11]. It is entirely possible that participants’ sleeping positions differed between sleeping in the laboratory and in their natural habitat.

## 5. Discussion

A relationship between the severity of OSA, as measured by the AHI, and positional dependency of sleep position was observed (cf. Figure 1, Table 1). Our study confirms that, in comparison to other sleep positions, the AHI was higher the longer the participant slept on their back (cf. Figure 2). Furthermore, our study results are in line with a disproportionately increasing AHI in overweight participants when in the supine position compared to those of normal-weight or underweight participants (cf. Figure 3).

The positional dependence of sleep apnea has been explored in various studies, and our findings are largely consistent with this body of research [14,18,19,22,24,25,40,41,42,43,44,45,46,47,48]. As can be seen in Figure 1, the boxplots of sleep position indicate that the AHI in the supine position deviates from that of the other positions, as previously demonstrated in other studies. Exploring the dependence on anatomical structures, Walsh, Jennifer et al. (2008) [49] found that the upper airways transition from a more elliptical shape in the supine position to a rounder shape in the lateral position, potentially increasing susceptibility to collapse due to increased permeability. Penzel et al. (2001) [41] pointed out that a lateral sleeping position might lead to the hypotonic genioglossus muscle inhibiting the tongue falling back, as well as to a reduction in the surrounding pressure within the tongue.

The supine position in patients can be avoided through relatively simple interventions, as some studies have investigated, such as using side-sleeper pillows, sewing tennis balls into nightwear, using electronic warning systems that alert users when they assume a supine position, or using a lightweight device with vibration in episodes of supine position, which should prompt the patient to change sleeping position [43,44,47]. This change alone may not eliminate OSA but presents a straightforward and cost-effective method for patients to improve their condition, highlighting the importance of a personalized approach to the diagnosis and treatment of OSA [50].

The interdependence of BMI and sleeping position is of more interest and has not been examined in a large population-based study before. In contrast to normal-weight and underweight individuals, Figure 3 elucidates an increase in the AHI with increasing time spent in the supine position during sleep among overweight participants. Notably, it exhibits a steeper incline. Similar to the current study, Itasaka et al. (2000) [25] observed a negative impact of a high BMI and supine sleeping position across all their variables, such as the AHI, like in our study, intraesophageal pressure, and oxygen saturation, which worsened with increasing BMI. Accordingly, they were able to observe improvements in all parameters when the sleep position was changed to lateral. Unlike our current study, Itasaka et al. [25] used a daytime PSG, where patients took sleeping medication beforehand and controlled their sleeping position for measuring the change from a supine to lateral sleeping position. In the study by Joosten et al. (2017) [26], a higher AHI in the supine position compared to the non-supine position was also affirmed. However, Joosten et al. (2017) [26] only included participants with overweight with an AHI over 20, as their emphasis was on the follow-up measurements after their patients experienced weight loss. With another approach, Oksenberg et al. (2012) [27] analyzed the longitudinal relationship between OSA, sleep position, AHI, and weight over a span of 6.2 years in 112 patients. They divided participants in positional patients (i.e., patients with OSA exhibiting over twice the frequency of breathing irregularities in the supine position compared to the lateral position) and non-positional patients. They observed that patients who lost weight substantially had a higher chance of becoming positional patients and vice versa, clearly demonstrating the connection between obesity and sleep position in the context of OSA. A link between OSA and pregnancy, with weight gain in pregnant women, was identified as a contributing factor [48].

Generally, patients with OSA can benefit substantially from weight loss [16,26]. Especially, the studies by Oksenberg et al. (2012) [27] and Joosten et al. (2017) [26] demonstrate the possibility of reducing the severity of OSA in the context of sleep position through weight loss. In a more recent study, the treatment of obesity with tirzepatide proved effective in improving OSA [51]. One might speculate that the parapharyngeal fat pads play an important role here. Pahkala et al. (2014) [16] found proportionally overlarge parapharyngeal fat pads in patients with OSA compared to weight-matched (but not age matched) habitual snorers. They also found that weight reduction reduced the AHI in those patients. It is conceivable that different sleep positions might mediate the impact of the parapharyngeal fat on the form and diameter of the pharynx. Further anatomical studies are needed to determine the interplay between fat localization and sleep position and its impact on sleep-disordered breathing (see Ivanovska et al., 2021 for a recent approach) [51].

## Figures and Tables

**Table 1:** Variables including Males, Females, supine sleep position, BMI, age, overweight, and normal or underweight in relation to the different severity levels of AHI.

	AHI < 5: Normal	5 ≤ AHI < 15: Mild	15 ≤ AHI < 30: Moderate	AHI ≥ 30: Severe
	*N* = 526	*N* = 267	*N* = 161	*N* = 91
Males [N, %]	232 (44%)	170 (64%)	108 (67%)	69 (76%)
Females [N, %]	294 (56%)	97 (36%)	53 (33%)	22 (24%)
Supine sleep position (% of total sleep time) [mean, SD]	49 (± 26.5)	47.6 (SD ± 26.8)	54.1 (± 28.3)	63.4 (± 28.1)
BMI (kg/m^2^) [mean, SD]	*M* = 26.5 (SD = 4.2)	*M* = 29.1 (SD = 4.5)	*M* = 30.8 (SD = 5.3)	*M* = 31.8 (SD = 4.9)
Age (years) [mean, SD]	*M* = 47 years (SD = 14)	*M* = 56 years (SD = 11)	*M* = 58 years (SD = 11)	*M* = 61 years (SD = 11)
Overweight (BMI ≥ 25) [N, %]	*N* = 324 (62%)	*N* = 226 (85%)	*N* = 145 (90%)	*N* = 87 (96%)
Normal weight and Underweight (BMI < 25) [N, %]	*N* = 202 (38%)	*N* = 41 (15%)	*N* = 16 (10%)	*N* = 4 (4%)

Note: SD: standard deviation; BMI: body mass index; overweight: people with a BMI ≥ 25, normal weight and underweight: people with a BMI < 25; see Supplementary Materials Table S1 for BMI and AHI in supine and non-supine positions.

## Data Availability

The data that support the findings of this study are available from the Transferstelle für Daten- und Biomaterialienmanagement (Office for transfer of data and biomaterials) of the University Medicine Greifswald, Study of Health in Pomerania (SHIP: https://transfer.ship-med.uni-greifswald.de/FAIRequest/login). Access is restricted and needs the approval of the board.

## Supplementary Materials

The following supporting information can be downloaded at: https://www.mdpi.com/article/10.3390/jpm14111087/s1, Table S1: BMI and AHI in supine and non-supine positions.
