# Supplementary material for: Association Between Sleep Position, Obesity, and Obstructive Sleep Apnea Severity
Source: J Pers Med. 2024 Nov 1;14(11):1087. doi: 10.3390/jpm14111087 (PMC11595661; doi:10.3390/jpm14111087)
Supplement: Supplementary file 1 [file jpm-14-01087-s001.zip › jpm-3207202-supplementary.pdf]

Table S1. BMI and AHI in supine and non-supine positions

| | Age | AHI supine | AHI prone | AHI left | AHI right |
|---|---|---|---|---|---|
| Overweight, *N* = 782 | M =55 years (SD =13) | M = 20.1 (SD = 22.6) | M = 6.2 (SD = 15.9) | M = 8.2 (SD = 16.2) | M = 5.1 (SD = 11.6) |
| Normal weight and Underweight, *N* = 263 | M =45 years (SD =15) | M = 6.6 (SD = 11.0) | M = 1.8 (SD = 6.2) | M =2.2 (SD = 6.2) | M = 1.4 (SD = 3.2) |

Note: Not all participants slept in all possible position; time spent in various position differ between participants; it should be considered that sleep positions might depend on age and weight.
